# Novel insights in pathophysiology of postoperative atrial fibrillation

**DOI:** 10.1016/j.xjon.2021.01.014

**Published:** 2021-03-09

**Authors:** Rohit K. Kharbanda, Mathijs S. van Schie, Yannick J.H. J. Taverne, Natasja M.S. de Groot, Ad J.J. C. Bogers

**Affiliations:** a Department of Cardiology, Erasmus Medical Center, Rotterdam, The Netherlands; b Department of Cardiothoracic Surgery, Erasmus Medical Center, Rotterdam, The Netherlands

**Keywords:** atrial fibrillation, electropathology, electrophysiology, cardiac mapping, AES, atrial extrasystoles, AF, atrial fibrillation, CB, conduction block, CD, conduction delay, EEA, endo-epicardial asynchrony, RA, right atrial/atrium

## Abstract

**Objectives:**

Atrial extrasystoles are usually benign; however, they can also trigger atrial fibrillation. It is most likely that if atrial extrasystoles provoke a larger amount of conduction disorders and a greater degree of endo-epicardial asynchrony, the risk of postoperative atrial fibrillation increases. To test this hypothesis, we investigated the effect of programmed atrial extrasystoles on endo-epicardial conduction and postoperative atrial fibrillation.

**Methods:**

Twelve patients (58% male, age 68 ± 7 years) underwent simultaneous endo-epicardial mapping (256 electrodes) of the right atrium during sinus rhythm and programmed atrial extrasystoles provoked from the right atrial free wall. Areas of conduction block were defined as conduction delays of ≥12 milliseconds and endo-epicardial asynchrony as activation time differences of exact opposite electrodes of ≥15 milliseconds.

**Results:**

Endo-epicardial mapping data of all programmed atrial extrasystoles were analyzed and compared with sinus rhythm (median preceding cycle length = 531 milliseconds [345-787] and median sinus rhythm cycle length = 843 milliseconds [701-992]). All programmed atrial extrasystoles were aberrant (severe, moderate, and mildly aberrant, respectively, n = 6, 3, and 3) and had a mean prematurity index of 50.1 ± 11.9%. The amount of endo-epicardial asynchrony (1% [1-2] vs 6.7 [2.7-16.9], *P* = .006) and conduction block (1.4% [0.6-2.6] vs 8.5% [4.2-10.4], *P* = .005) both increased during programmed atrial extrasystoles. Interestingly, conduction block during programmed atrial extrasystoles was more severe in patients (n = 4, 33.3%) who developed postoperative atrial fibrillation (5.1% [2.9-8.8] vs 11.3% [10.1-12.1], *P* = .004).

**Conclusions:**

Atrial conduction disorders and endo-epicardial asynchrony, which play an important role in arrhythmogenesis, are enhanced during programmed atrial extrasystoles compared with sinus rhythm. The findings of this pilot study provide a possible explanation for enhanced vulnerability for postoperative atrial extrasystoles to induce postoperative atrial fibrillation in patients after cardiac surgery.

## Graphical abstract

*Left panel*: This pilot study revealed that P-AES originating from the free wall of the RA (1) provoked a substantial increase in endo- and epicardial conduction disorders, (2) enhanced electrical asynchrony between both layers up to 44 milliseconds, and (3) provoked more conduction disorders and EEA in patients who developed POAF compared with patients who remained postoperatively in SR. *Right panel*: Typical endo-epicardial activation maps of a sinus rhythm beat (*left*) and programmed AES (*right*) obtained from the same patient are shown. *Black arrows* display the main trajectories of the electrical wavefronts and local activation times are depicted at the head and tail. *Thick black lines* indicate lines of conduction block. Enhanced electrical disturbances can be observed during programmed AES compared with sinus rhythm. These findings may explain why postoperative AES may induce POAF. *P-AES*, Programmed atrial extrasystoles; *RA*, right atrium; *EEA*, endo-epicardial asynchrony; *POAF*, postoperative atrial fibrillation; *SR*, sinus rhythm; *AES*, atrial extrasystoles.

**Figure:**
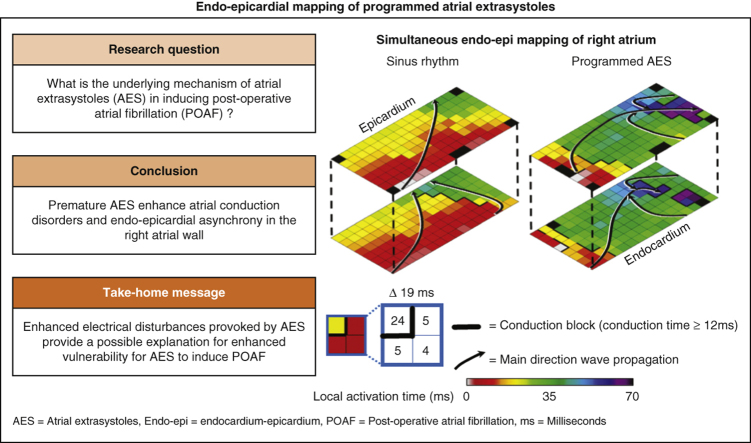

**Figure:**
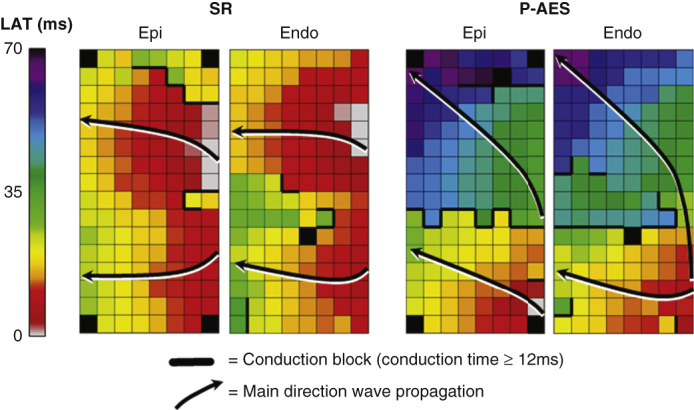
Programmed atrial extrasystoles enhance endo- epicardial conduction disorders.

**Central Message**

Programmed atrial extrasystoles (AES) enhance endo-epicardial asynchrony and conduction disorders, which may explain enhanced vulnerability for AES to induce postoperative atrial fibrillation.

**Perspective**

Simultaneous endo-epicardial mapping of the right atrium revealed a significant increase in endo-epicardial conduction disorders and asynchrony during programmed atrial extrasystoles (AES) compared with sinus rhythm, which may furnish intramural reentry. This pilot study provides a possible explanation for enhanced vulnerability for postoperative AES to induce postoperative atrial fibrillation.

See Commentaries on pages 130 and 131.

It is well known that postoperative atrial fibrillation (AF) is the most common complication following cardiac surgery and is associated with a prolonged hospital stay and increased risk of other complications, such as hemodynamic instability and thromboembolic events.^1^ The underlying mechanism of postoperative AF is considered to be a multifactorial combination of pre-existing (eg, age and cardiovascular risk factors), intraoperative (eg, type of surgery), and postoperative factors such as inflammation and enhanced sympathetic activity. The inflammatory response and increased sympathetic activity associated with cardiopulmonary bypass during cardiac surgery enhances automaticity and may provoke atrial extrasystoles (AES).^2^ As shown in several studies, in the vast majority of the patients, postoperative AF episodes are preceded by AES.^3^, ^4^, ^5^ Mapping studies have shown that AES enhance conduction disorders and endo-epicardial asynchrony (EEA), which play an important role in the pathophysiology of AF.^6^^,^^7^ In these mapping studies, only spontaneous AES were investigated, but the origin of the AES could not be retrieved.

Already more than 2 decades ago, Papageorgiou and colleagues demonstrated that high right atrial (RA) stimulation provoked more conduction disorders compared with stimulation from the coronary sinus. The authors postulated that the junctional area of the anisotropic crista terminalis and ramification of the pectinate muscles favors nonuniform slow atrial conduction and therefore may explain their observations.^8^^,^^9^ It is most likely that if AES provoke a larger amount of conduction disorders and a greater degree of EEA, the risk of postoperative AF increases. In this pilot study, to test this hypothesis, we investigated the effect of programmed high RA stimulation, thereby mimicking the impact of spontaneous AES, on endo- and epicardial conduction and postoperative AF.

## Methods

### Aim

We aimed to (1) unravel the effect of programmed AES on endo-epicardial conduction in the right atrial wall and to (2) correlate this mapping data with the incidence of postoperative AF. This pilot trial is undertaken to provide sufficient assurance to enable a larger trial to further investigate our hypothesis.

### Study Population

Simultaneous endo-epicardial mapping was performed in patients undergoing elective primary open-heart surgery in the Erasmus Medical Center Rotterdam. Patients with hemodynamic instability, atrial pacing, previous cardiac surgery, or severely impaired left ventricular function (ejection fraction <30%) were excluded. Patients underwent either coronary artery bypass surgery, valve surgery, or a combination of both. This pilot study was approved by the institutional medical ethical committee (MEC2015-373), and written informed consent was obtained from all participants. Patient characteristics were obtained from electronic medical files. The study was carried out according to the principles of the Declaration of Helsinki.

### Simultaneous Endo-Epicardial Mapping of the RA

**Figure 1:**
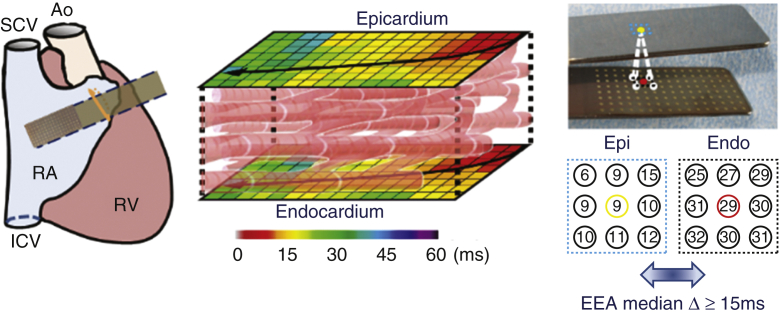
Simultaneous endo-epicardial mapping of the RA was performed using two 128-electrode arrays secured exactly opposite of each other on 2 spatulas (*left panel*). Color-coded activation maps of the endo- and epicardium are shown in the *middle panel*. *Black arrows* display the main trajectories of the electrical wavefronts. To calculate EEA, for each electrode, the median time delay within the exact opposite electrode and its eight surrounding electrodes was selected. The longest time delay for every endo-epicardial electrode pair is then selected to express local degree of EEA, defined as transmural difference in electrical activation of ≥15 milliseconds between every endo-epicardial electrode pair. *Ao*, Aorta; *SCV*, superior caval vein; *RA*, right atrium; *RV*, right ventricle; *ICV*, inferior caval vein; *Epi*, epicardium; *Endo*, endocardium; *EEA*, endo-epicardial asynchrony.

Simultaneous endo-epicardial high-density and resolution mapping of the RA was performed before the commencement of extracorporeal circulation, as previously described in detail.^10^ Two multielectrode arrays, each containing 128 electrodes with a diameter of 0.45 mm and with 2 mm interelectrode spacing, were attached on 2 bendable spatulas and positioned on the exact opposite side of each other. A temporary bipolar pacemaker wire was stitched to the free wall of the RA serving as a temporal reference electrode. The indifferent electrode was connected to a steel wire stitched in the subcutaneous tissue. The endocardial electrode array was introduced in the RA in the auricular purse string suture for the venous cannula. Simultaneous endo-epicardial mapping of the mid-RA was performed, as depicted in the left panel of Figure 1. Simultaneous endo-epicardial mapping was performed during sinus rhythm (SR) followed by programmed electrical stimulation at the RA free wall. Recorded data included a surface electrocardiogram lead, calibration signal of 2 mV and 1000 milliseconds, bipolar reference electrogram, and 253 endo- and epicardial unipolar electrograms. Recordings were analog-to-digital converted (16-bits), sampled with a rate of 1 kHz, amplified (gain 1000), and filtered (bandwidth 0.5-400 Hz).

### Mapping Data Analysis

Custom-made software was used to analyze the mapping data as previously described in detail.^10^ Color-coded activation maps of both the endo- and epicardial layer were constructed by annotating the steepest negative slope of atrial potentials recorded at every electrode.

### Atrial Conduction Disorders

Consistent with previous mapping studies, areas of conduction delay (CD) and conduction block (CB) were defined as interelectrode differences in local activation times of, respectively, 7-11 milliseconds and ≥12 milliseconds.^10^ These cutoff values correspond with effective conduction velocities of, respectively, 18 to 28 cm/s for CD and <18 cm/s for CB. Areas of uninterrupted CD and CB lines were defined as continuous CDCB lines. In addition, the median and maximal length of all CB and continuous CDCB lines were calculated.

### Endo-epicardial Asynchrony

As demonstrated in the middle and right panel of Figure 1, local endo-epicardial activation time differences were determined by selecting the median of the time delays within the exact opposite electrode and its 8 surrounding electrodes. The longest time delay for every endo-epicardial electrode pair is then selected for the asynchrony map demonstrating local degree of EEA. Consistent with previous studies, EEA was defined as transmural difference in electrical activation of ≥15 milliseconds between every endo-epicardial electrode pair.^10^^,^^11^

### Classification of Programmed AES

Prematurity index of programmed AES was expressed as the ratio between the coupling interval of the programmed AES and the preceding SR cycle length. In general, we aimed to achieve a mean prematurity index of approximately 50%. Depending on the degree of shift in wavefront direction during programmed electrical stimulation compared with SR, patterns of activation during programmed AES were classified as mildly, moderately, or severely aberrant (respectively, 0-45°, 135-180°, or 90° shift).^6^^,^^7^

### Statistical Analysis

Normally distributed continuous variables are expressed as mean ± standard deviation and skewed variables as median with interquartile range. Categorical data are presented as numbers and percentages and compared with the χ^2^ test. Comparison of conduction disorders between the endo- and epicardium was performed with the Wilcoxon signed rank test. Association of clinical characteristics and electrophysiological parameters were analyzed with the Wilcoxon rank-sum test. Statistical analyses were performed using IBM SPSS Statistics, version 21 (IBM Corp, Armonk, NY).

## Results

### Study Population

Baseline characteristics of the 12 enrolled patients (68 ± 7 years, 7 male) are summarized in Table 1. One half of the patients had ischemic heart disease (n = 6) and 4 (33.3%) had a history of paroxysmal AF. Seven patients underwent coronary artery bypass grafting (n = 1, concomitant mitral valve repair), 4 patients underwent aortic valve replacement (n = 1 concomitant mitral valve repair and n = 1 concomitant mitral and tricuspid valve repair), and 1 patient underwent a Cox-maze IV procedure. None of the patients had dilated RA, and the majority had normal left ventricular function (n = 7, 58.3%). Postoperative AF occurred in 4 patients (33.3%) of whom 2 with new-onset postoperative AF.

**Table 1:** Patient characteristics

Number of patients	12
Age, y	68 ± 7 [51-78]
Male	7 (58.3)
Underlying heart disease	n (%)
IHD	6 (50)
VHD	4 (33.3)
I/VHD	1 (8.3)
Lone AF	1 (8.3)
Surgical procedure	n (%)
CABG	6 (50)
CABG + MVR	1 (8.3)
AVR	2 (16.6)
AVR + MVR	1 (8.3)
AVR + MVR + TVR	1 (8.3)
Cox-Maze IV	1 (8.3)
History of AF	
Paroxysmal	4 (33.3)
Cardiovascular risk factors	
BMI, kg/m^2^	28.6 ± 3.8 [23.7-36.3]
Hypertension	8 (66.7)
Dyslipidemia	4 (33.3)
Diabetes mellitus	4 (33.3)
Left ventricular function	
Normal	7 (58.3)
Mild/moderate dysfunction	5 (41.7)

Numbers in brackets are ranges. *IHD*, Ischemic heart disease; *VHD*, valvular heart disease; *I/VHD*, ischemic and valvular heart disease; *AF*, atrial fibrillation; *CABG*, coronary artery bypass grafting; *MVR*, mitral valve repair; *AVR*, aortic valve replacement; *TVR*, tricuspid valve repair; *BMI*, body mass index.

### Characteristics of Programmed AES

All programmed AES were aberrant (6 severely, 3 moderately, and 3 mildly aberrant) and had a mean prematurity index of 50.1 ± 11.9%. The preceding median coupling interval and median cycle length during SR recording were, respectively, 531 milliseconds [345-787] and 843 milliseconds [701-992].

### Endo- and Epicardial Conduction Disorders

Table 2 summarizes the amount and extensiveness of conduction disorders during both SR and programmed AES. During SR, the total amount of CB and continuous CDCB observed in both the endo- and epicardium together was 1.4% [0.6-2.6] and 1.9% [0.9-4.1], respectively. This resulted in a median length of 4 mm [4-6] for CB and 8 mm [6.5-12] for continuous CDCB. There were no differences observed in amount and extensiveness of conduction disorders between the endo- and epicardial layer separately during SR (Table E1, all *P* > .156).

**Table 2:** Characteristics of conduction disorders during sinus rhythm and programmed AES

	SR	P-AES	*P* value
% CB	1.4 [0.6-2.6]	8.5 [4.2-10.4]	**.005**
Length CB lines	4 [4-6]	4.5 [4-6]	.570
Max length CB lines	10 [6.5-16.5]	21 [15-25]	**.026**
% Continuous CDCB	1.9 [0.9-4.1]	10.7 [5.7-15.1]	**004**
Length continuous CDCB	8 [6.5-12]	11 [8.3-20]	.239
Maximal length continuous CDCB	10 [11-29]	28 [16.5-35.5]	.196
Maximal conduction time	19.5 [15-29.8]	29.5 [23-37.8]	.059
% EEA	1 [1-2]	6.7 [2.7-16.9]	**.006**
Median endo-epicardial delay	16.5 [0-19]	20 [16.3-20.8]	.050
Maximal endo-epicardial delay	18.3 [0-25.8]	24 [19.5-33.9]	**.036**

Length of lines is expressed in millimeters and conduction time in milliseconds. *P* values below .05 as statistically significant indicated in bold. *SR*, Sinus rhythm; *P-AES*, programmed atrial extra systoles; *CB*, conduction block; *CDCB*, conduction delay-conduction block; *EEA*, endo-epicardial asynchrony.

**Figure 2:**
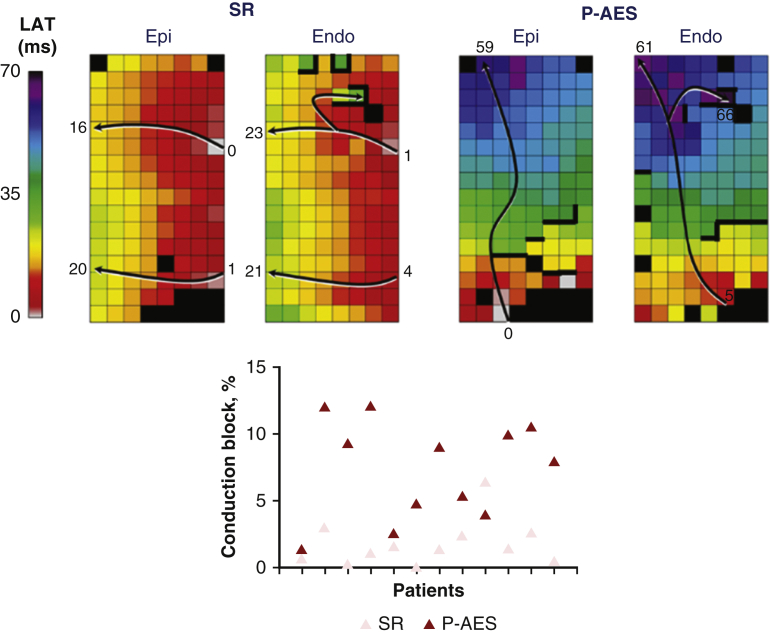
*Upper panel*: Typical endo-epicardial activation maps of a SR beat (*left*) and P-AES (*right*) obtained from the same patient. *Black arrows* display the main trajectories of the electrical wavefronts and local activation times are depicted at its head and tail. *Thick black lines* indicate lines of conduction block. The *lower panel* shows the effect of programmed right atrial stimulation on the amount of conduction block (*y-axis*) for each patient (*x-axis*) separately. Increase in total activation time and amount of conduction block is observed during P-AES compared with SR. *SR*, Sinus rhythm; *LAT*, local activation times; *Epi*, epicardium, *Endo*, endocardium; *P-AES*, programmed atrial extrasystoles.

The upper panel of Figure 2 shows typical endo-epicardial activation maps of one single SR beat and programmed AES obtained from the same patient. A substantial increase in CB, indicated by thick black lines, is demonstrated during programmed AES compared with SR. The lower panel of Figure 2 shows the effect of programmed atrial stimulation on the amount of CB for each patient separately. During programmed AES, the total amount of CB and continuous CDCB in both layers together significantly increased from 1.4% [0.6-2.6] to 8.5% [4.2-10.4] and from 1.9% [0.9-4.1] to 10.7% [5.7-15.1] respectively (both *P* ≤ .005). Characteristics of conduction disorders during programmed AES did not differ between the endo- and epicardial layer separately (Table E1, all *P* > .167), except for a greater maximal conduction time at the endocardium (29.5 milliseconds [18.5-37.5] vs 23 milliseconds [16.3-23], *P* = .033).

### Endo-Epicardial Asynchrony

**Figure 3:**
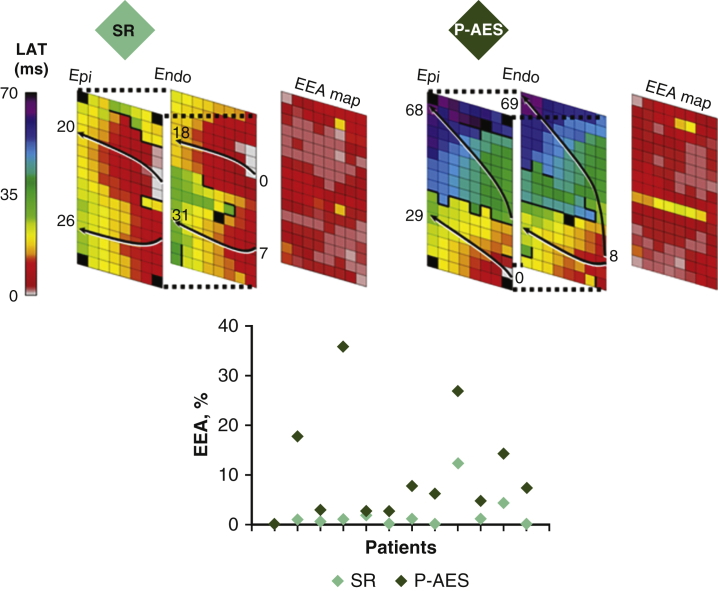
*Upper panel*: Endo-epicardial activation maps and corresponding EEA maps of one single SR beat (*lef*t) and P-AES (*right*) obtained from the same patient. *Black arrows* display the main trajectories of the electrical wavefronts and local activation times are depicted at its head and tail. *Thick black lines* indicate lines of conduction block. The *lower panel* shows the effect of programmed right atrial stimulation on the amount of EEA for each patient separately. An increase in conduction block and EEA is observed during P-AES compared with SR. *SR*, Sinus rhythm; *LAT*, local activation times; *Epi*, epicardium; *Endo*, endocardium; *EEA*, endo-epicardial asynchrony; *P-AES*, programmed atrial extrasystoles.

In the upper panel of Figure 3, endo-epicardial activations maps and corresponding EEA maps demonstrate the incremental effect of programmed AES on electrical asynchrony between both atrial layers. In the lower panel of Figure 3, differences in amount of EEA during SR (light green) and programmed AES (dark green) are shown for each patient separately. Overall, programmed AES provoked a significant increase in EEA from 1% [1-2] during SR to 6.7% [2.7-16.9] during programmed AES (*P* = .006). The severity of EEA during programmed AES, expressed in median and maximal endo-epicardial delay, also increased from 16.5 to 20 milliseconds (*P* = .05) and 18.3 to 24 ms (*P* = .036), respectively.

### Postoperative AF

Differences in characteristics of programmed AES between patients with and without postoperative AF are summarized in Table 3. Programmed AES caused significantly more conduction disorders and EEA in patients who developed postoperative AF compared with patients without postoperative AF. Not only was the prevalence of CB (5.1% [2.9-8.8] vs 11.3% [10.1-12.1], *P* = .004) and continuous CDCB (6.7% [5.2-11.6] vs 16% [14.1-18.5], *P* = .004) significantly greater in patients with postoperative AF, but also the length of continuous CDCB lines (9.5 mm [8-11] vs 22 mm [15.3-23.5], *P* = .008).

**Table 3:** Differences in characteristics of programmed AES between patients with and without POAF

	No POAF (n = 8)	POAF (n = 4)	*P* value
% CB	5.1 [2.9-8.8]	11.3 [10.1-12.1]	**.004**
Length CB lines	4 [2.5-5.5]	5.5 [5-7.5]	.073
Max length CB lines	21 [9.5-22]	22 [18-33.5]	.368
% Continuous CDCB	6.7 [5.2-11.6]	16 [14.1-18.5]	**.004**
Length continuous CDCB	9.5 [8-11]	22 [15.3-23.5]	**.008**
Maximal length continuous CDCB	21 [14.5-31.5]	42 [28.5-57]	**.028**
Maximal conduction time	24.5 [18.5-29.8]	38.5 [37.3-47.3]	**.004**
% EEA	4.5 [2.6-7.6]	16.0 [7.1-31.4]	.109
Median endo-epicardial delay	17.3 [15.6-20]	22 [20-24.8]	**.048**
Maximal endo-epicardial delay	22 [16.8-24]	36 [27-42]	**.016**

Length of lines is expressed in millimeters and conduction time in milliseconds. *P* values below .05 as statistically significant indicated in bold. *POAF*, Postoperative atrial fibrillation; *CB*, conduction block; *CDCB*, conduction delay-conduction block; *EEA*, endo-epicardial asynchrony.

Despite the clinically relevant EEA provoked by programmed AES in patients who encountered postoperative AF (4.5% vs 16%), it did not reach statistical significance (*P* = .109). Median and maximal endo-epicardial delay measured between both atrial layers, however, was significantly greater in patients with postoperative AF (respectively, *P* = .048 and *P* = .016). Cardiopulmonary bypass- and aortic crossclamp time were similar between both groups (both *P* > .109).

## Discussion

### Key Findings

**Figure 4:**
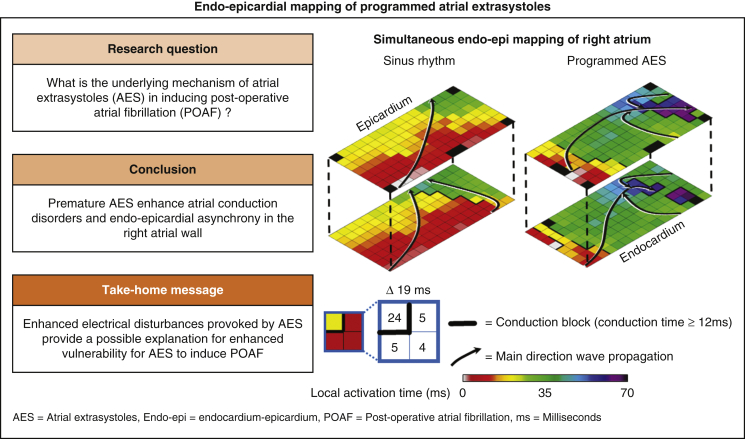
*Left panel*: This pilot study revealed that P-AES originating from the free wall of the RA (1) provoked a substantial increase in endo- and epicardial conduction disorders, (2) enhanced electrical asynchrony between both layers up to 44 milliseconds, and (3) provoked more conduction disorders and EEA in patients who developed POAF compared with patients who remained postoperatively in SR. *Right panel*: Typical endo-epicardial activation maps of a sinus rhythm beat (*left*) and programmed AES (*right*) obtained from the same patient are shown. *Black arrows* display the main trajectories of the electrical wavefronts and local activation times are depicted at the head and tail. *Thick black lines* indicate lines of conduction block. Enhanced electrical disturbances can be observed during programmed AES compared with sinus rhythm. These findings may explain why postoperative AES may induce POAF.

Simultaneous endo-epicardial mapping of the RA during SR and programmed RA stimulation revealed that programmed AES originating from the free wall of the RA (1) provoked a substantial increase in endo- and epicardial conduction disorders, (2) enhanced electrical asynchrony between both layers up to 44 milliseconds and covering 36% of the mapping area and (3) provoked more conduction disorders and EEA in patients who developed postoperative AF compared with patients who remained postoperatively in SR. The findings of this pilot study provide a possible explanation for enhanced vulnerability for postoperative AES to induce postoperative AF in patients after cardiac surgery and are summarized in Figure 4.

### Effect of (Programmed) Right AES on Endo-Epicardial Conduction

Previous intraoperative mapping studies examined the effect of spontaneous AES on atrial conduction and showed an increase in epicardial conduction disorders, especially during aberrant AES.^6^ Simultaneous endo-epicardial mapping of the RA during spontaneous AES revealed that this increase in conduction disorders may be unequally expressed between the endo- and epicardium giving rise to EEA.^7^ An important limitation of these studies is lack of information on the origin of the AES. To overcome this limitation, in the present study, programmed premature atrial stimulation was performed at the RA free wall as we assume that excessive surgical manipulation in combination with surgical incisions in the RA appendage and purse-string suture induced local ischemia, are likely to provoke AES originating from the RA free wall in patients after cardiac surgery. In addition, enhanced anisotropic properties of the RA tissue may reinforce conduction disorders and EEA during AES thereby increasing the vulnerability of the atria for arrhythmias. AES that provoke a larger amount of conduction disorders and a higher degree of EEA are more likely to induce postoperative AF episodes, which is supported by the present pilot study showing more pronounced conduction disorders during programmed AES in patients who developed postoperative AF compared with patients who remained in SR.

It is generally assumed that the complex architecture and anatomy of both atria play an important role in arrhythmogenesis. Fiber orientation and atrial wall thickness may vary between different atrial regions. Even at one specific site, differences in fiber orientation between the endo- and epicardium may be observed. Any premature AES originating from an anisotropic region may provoke significant conduction disorders that may affect the endo- and epicardium unequally. This may provoke or aggravate EEA in the atrial wall, thereby possibly initiating transmural reentry. Spach and colleagues^12^ demonstrated that programmed premature atrial stimulation in isolated anisotropic muscle fibers resulted in dissociated conduction that causes unidirectional CB, thereby providing a potential substrate for re-entry.

Schuessler and colleagues^13^ have investigated endo-epicardial conduction at the RA appendage of canines during SR and programmed premature atrial stimulation. Endo-epicardial asynchrony was greater during programmed AES with a greater prematurity index and increased mostly with prematurely aberrant AES, which is in line with previous studies from our group.^6^^,^^7^ Premature AES provoked EEA up to 30 milliseconds. Subsequently, they induced tachyarrhythmia by an extra-stimulus during intravenous acetylcholine administration. A 3-dimensional pathway using a free-running muscle bundle between the endo- and epicardium was part of the induced reentry circuit with a cycle length as short as approximately 60 milliseconds. Recently, endo-epicardial optical mapping combined with high-resolution 3-dimensional gadolinium-enhanced magnetic resonance imaging (80 μm^3^ resolution) demonstrated similar re-entry loops using transmural pectinate muscles in ex vivo human RA.^14^ These findings provide a possible explanation for the underlying mechanism of postoperative AES inducing episodes of postoperative AF.

### Factors Contributing to Enhanced Arrhythmogenicity of AES

The arrhythmogenicity of an AES is determined by AES-related characteristics (eg, prematurity), anatomical features (eg, 3-dimensional fiber orientation, fibrosis), and alterations in the cardiac autonomic tone (eg, enhanced sympathetic or parasympathetic activity).

Refractory periods of adjacent cardiomyocytes may vary and are influenced by several factors, such as heart rate and autonomic tone. When the prematurity of AES increases, it is more likely that the electrical wavefront encounters areas with differences in excitability of atrial tissue which may promote development of AF.^12^ This effect may be enhanced by changes in autonomic tone. As the cardiac autonomic nervous system is heterogeneously distributed between both atria, changes in sympathetic and parasympathetic tone may enhance dispersion of atrial refractoriness and promote differences in local conduction velocities. It is generally believed that, enhanced sympathetic tone increases calcium loading, thereby enhancing triggered activity, while enhanced parasympathetic tone slows conduction velocity, shortens the refractory period and increases dispersion of atrial refractoriness, thereby facilitating re-entry.^15^

Conduction velocity is also dependent on the 3-dimensional anatomical features of the area where the AES originates.^16^ To initiate an endo-epicardial reentry circuit at an area with significant EEA, transmural muscle fibers connecting the endo- and epicardium are a prerequisite. This 3-dimensional architecture can also be disrupted by structural remodeling caused by ageing, coronary- or valvular heart disease or tachyarrhythmia-induced electrical remodeling.^17^, ^18^, ^19^ Furthermore, when a small atrial muscle bundle has to excite a relatively large heterogeneous area, sink-to-source mismatch may occur resulting in slowing of atrial conduction and breaking of wavefronts, which may initiate re-entry.^16^

### Premature AES After Cardiac Surgery, Common or Not?

The relationship between frequent AES and greater incidence of AF has been demonstrated in different populations.^20^^,^^21^ There are only a few studies investigating the role of premature AES in initiation of postoperative AF after cardiac surgery. After Frost and colleagues^22^ had demonstrated that premature AES could initiate postoperative AF, several studies demonstrated that the incidence of postoperative AF could be reduced by atrial overdrive pacing thereby suppressing atrial premature depolarizations.^3^^,^^23^, ^24^, ^25^, ^26^ Blommaert and colleagues introduced an algorithm for dynamic overdrive pacing which reacts to premature AES by increasing its frequency.^3^ A significant reduction in the incidence of postoperative AF was observed in the pacing group compared to controls (10% vs 27% respectively, *P* = .036). The aforementioned studies evaluated the performance of atrial overdrive pacing suppressing premature AES to prevent postoperative AF. However, there are only a few studies investigating the incidence of premature AES in relation to postoperative AF.^5^^,^^27^, ^28^, ^29^ Jidéus and colleagues demonstrated that in patients who developed postoperative AF after coronary artery bypass grafting, it was initiated by premature AES in 81% of the patients.^5^ Previously, the same research group already demonstrated that a greater preoperative burden of premature AES is associated with a greater risk of postoperative AF.^28^ Recently, Hashimoto and colleagues^27^ observed significantly more premature AES/24 hours in patients who developed new-onset postoperative AF compared with patients who remained in SR (4128 ± 7186 vs 69 ± 221, *P* < .001) after off-pump coronary artery bypass. In line with the findings of Jidéus and colleagues^28^ and Frost and colleagues,^29^ frequent premature AES (>47 per 24 hours) appeared to be a predictor for postoperative AF. Yaksh and colleagues^30^ were the first to determine whether postoperative AES burden is associated with postoperative AF using postoperative telemetry data of 29 patients with postoperative AF and controls. Patients with postoperative AF showed a greater burden of premature AES compared with controls (0.9% vs 0.2%, *P* = .001). Moreover, AES triggering AF episodes were more often premature (*P* < .001).

### Limitations

This proof-of-concept study is mainly limited by the low sample size, and the results should therefore be interpreted with caution. Future larger studies are required to substantiate our findings. We could not address whether AES also enhance electrical disturbances in the left atrium. Due to safety reasons, simultaneous endo-epicardial mapping of the left atrium can only be performed under specific circumstances and was therefore not performed in the present pilot study. Moreover, to determine the effect of AES on endo-epicardial conduction in both atria, total simultaneous endo-epicardial mapping should be performed which is technically impossible. Future studies focusing on both atria are required to further unravel the arrhythmogenic effects of AES originating from different sites.

## Conclusions

The exact mechanistic role of AES in initiating postoperative AF is unknown. In the present pilot study, premature programmed AES at the RA aggravated endo-epicardial conduction disorders and electrical asynchrony between both layers occurring up to 44 milliseconds and covering 36% of the mapping area (Video 1). Enhanced conduction disorders and EEA provoked by premature AES are potential mechanisms for intramural re-entry, which may result in postoperative AF. Larger studies are needed to assess whether intraoperative cardiac mapping including programmed atrial stimulation may predict development of postoperative AF.

### Conflict of Interest Statement

The authors reported no conflicts of interest.

The *Journal* policy requires editors and reviewers to disclose conflicts of interest and to decline handling or reviewing manuscripts for which they may have a conflict of interest. The editors and reviewers of this article have no conflicts of interest.

## Figures and Tables

**Table E1:** Differences in conduction disorders between the endo- and epicardium

	SR			P-AES		
	Endo	Epi	*P* value	Endo	Epi	*P* value
% CB	1.6 [0.5-2.9]	0.9 [0.1-2.4]	.239	7.7 [3.7-11.0]	7.7 [2.5-9.3]	.433
Length CB lines	4 [3.3-7.5]	2 [0.5-4.8]	.156	6 [4-10.3]	3.5 [2.3-5.5]	.213
Max length CB lines	9 [5-11.5]	5 [1-11.5]	.167	19 [8.5-22]	13 [5.5-18]	.167
% Continuous CDCB	2.3 [0.6-3.6]	2.1 [0.1-3.7]	.875	8.6 [5.8-16.5]	9.9 [5.9-16.5]	.638
Length continuous CDCB	8 [8-12.3]	8 [1-12.8]	.529	15.5 [8.3-21.5]	11.5 [8.5-14.5]	.328
Max length continuous CDCB	11 [8.5-29]	14 [1-21]	.694	24 [16-31.5]	26 [14-31.5]	.362
Maximal conduction time	17.5 [15-25.8]	15 [11.8-25.8]	.610	29.5 [18.5-37.5]	23 [16.3-31]	**.033**

Length of lines is expressed in millimeters and conduction time in milliseconds. *P* values below .05 as statistically significant indicated in bold. *SR*, Sinus rhythm; *P-AES*, programmed atrial extra systoles; *Endo*, endocardium; *Epi*, epicardium; *CB*, conduction block; *CDCB*, conduction delay-conduction block.

## Supplementary Data

Video 1 Dr Kharbanda provides a summary of the purpose, methods, results and limitations of this study. Video available at: https://www.jtcvs.org/article/S2666-2736(21)00054-1/fulltext.
