# Supplementary figures and images for: Novel insights in pathophysiology of postoperative atrial fibrillation
Source: JTCVS Open. 2021 Mar 9;6:120–9. doi: 10.1016/j.xjon.2021.01.014 (PMC9390318; doi:10.1016/j.xjon.2021.01.014)

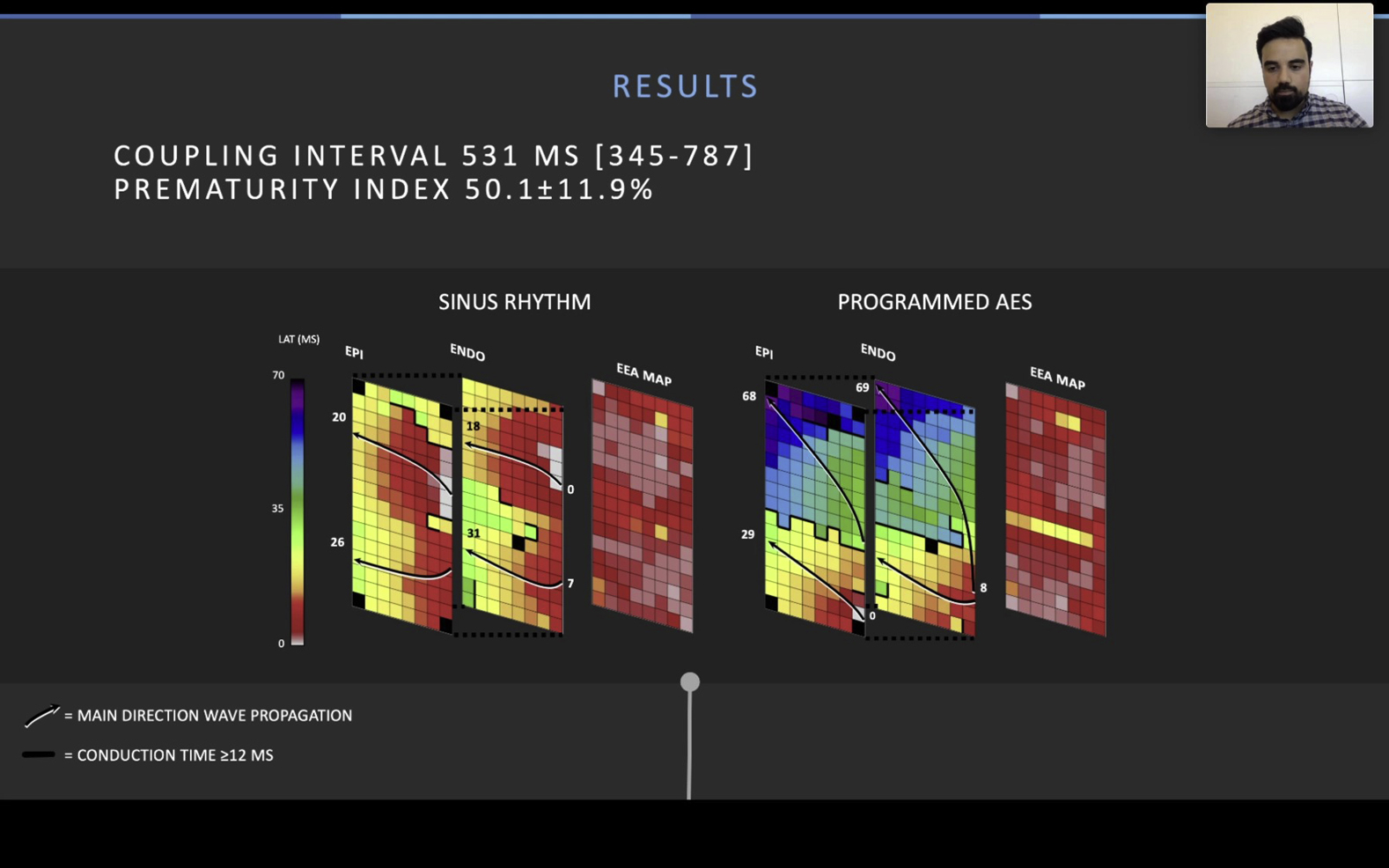

Supplement: Video 1 — Dr Kharbanda provides a summary of the purpose, methods, results and limitations of this study. Video available at: https://www.jtcvs.org/article/S2666-2736(21)00054-1/fulltext. [file fx3.jpg]
